# Alterations in lipid profile in neonatal calves affected by diarrhea

**DOI:** 10.14202/vetworld.2017.786-789

**Published:** 2017-07-16

**Authors:** K. Bozukluhan, O. Merhan, H. I. Gokce, H. A. Deveci, G. Gokce, M. Ogun, S. Marasli

**Affiliations:** 1 Kars School of Higher Vocational Education, University of Kafkas, Kars, Turkey; 2 Department of Biochemistry, Faculty of Veterinary Medicine, University of Kafkas, Kars, Turkey; 3 Department of Internal Medicine, Faculty of Veterinary Medicine, University of Mehmet Akif Ersoy, Burdur, Turkey; 4 Islahiye School of Higher Vocational Education, University of Gaziantep, Gaziantep, Turkey; 5 Department of Internal Medicine, Faculty of Veterinary Medicine, University of Kafkas, Kars, Turkey

**Keywords:** calves, cholesterol, diarrhea, high-density lipoprotein-cholesterol, very low-density lipoprotein-cholesterol, triglycerides

## Abstract

**Aim:**

The aim of this study was to determine the alterations in the lipid profile, plasma alkaline phosphatase (ALP) activity, total and direct bilirubin levels of neonatal calves with diarrhea.

**Materials and Methods:**

A total of 25 calves with diarrhea as experimental group and 10 healthy calves as control group, 1-30 days old, were enrolled in the study. Blood samples were collected from jugular vein in tubes with anticoagulant agent to evaluate the concentration of triglycerides, total cholesterol, high-density lipoprotein-cholesterol (HDL-C), ALP, total and direct bilirubin. Very low-density lipoprotein-cholesterol (VLDL-C) and low-density lipoprotein-cholesterol (LDL-C) levels were calculated according to the Friedewald formula.

**Results:**

Significant increases in the plasma levels of ALP (p<0.05), total and direct bilirubin, triglycerides, and VLDL-C (p<0.01) were determined, whereas significant decreases in the levels of total cholesterol, HDL-C and LDL-C (p<0.01) were observed in neonatal calves with diarrhea.

**Conclusion:**

According to the findings of this study, liver functions impaired and, therefore, lipid profile is affected negatively in neonatal calves with diarrhea.

## Introduction

The neonatal period represents a critical stage in the development of physiological functions. During this phase, the newborn has to face several adjustments to adapt the body systems to extrauterine life [1]. Newborns are in metabolically unstable conditions, which make these subjects particularly sensitive to perinatal diseases resulting in high mortality rates [1,2].

Neonatal diarrhea is a one of the major causes of morbidity and mortality in calves during the first 3 weeks of life, resulting in considerable economic loss. Calf diarrhea is mainly caused by several pathogenic microorganisms such as *Escherichia coli*, *Salmonella*, *Rotavirus*, *Coronavirus*, and *Cryptosporidium* in association with environmental factors, animal’s immunological structure and nutritional status [3,4]. Calves are particularly susceptible to diarrhea caused by enteropathogens in neonatal period, especially if they have failure of passive transfer of immunoglobulins [5-7].

Bovine placenta does not permit the passive transfer of antibody to the fetus. After bird, the immune system is not fully developed, and the production of endogenous antibody does not rich protective levels until 1 month of age. Therefore, resistance of newborn calf to enteropathogens depends on an adequate passive transfer of colostral immunoglobulins. Failure of passive transfer predisposes neonates to infection and septicemia and often results in severe and fatal disease [4,8,9].

In diarrhea, metabolic changes such as dehydration, metabolic acidosis, electrolyte imbalance, negative energy balance, and/or hypoglycemia occur at various degrees depending on the severity of the infection. The severity of the metabolic alterations significantly influences the survivability of diarrheic neonates [10,11].

In case of negative energy balance and/or disease, animal has to provide the majority of energy via lipolysis. On the other hand, lipids play an important role on synthesis of several organic substances together with carbohydrates and proteins. They have also used in biomembrane structures and as hormone precursors [12]. Thus, changes in plasma lipid levels affect other organ or tissue functions [12,13].

The lipids are stored as triglycerides and transported as fatty acid bound to albumin in the blood. The majority of these fatty acids in the blood are metabolized into carbon dioxide and/or ketone bodies by the liver. The remaining, cholesterol, is combined with phospholipid and released into the blood as very low-density lipoprotein-cholesterol (VLDL-C) [14,15]. The degradation of VLDL-C results in the production of low-density lipoprotein-cholesterol (LDL-C) that carries cholesterol to peripheral tissues, while high-density lipoprotein-cholesterol (HDL-C) that transfers cholesterol from peripheral tissues to the liver is produced from degradation of LDL-C [13,14].

It has been reported that infections and inflammations impair lipid and lipoprotein metabolism, like lipid oxidation and cholesterol transport to stimulate anti-inflammatory response [16-18]. During the infection or endotoxemia, a systemic inflammatory response develops and, if it is severe enough, it may damage and impair several organ or systems functions such as liver, kidney, respiratory, and circulatory system [19-21]. Depending on organ or system dysfunction, several metabolic disorders such as metabolic acidosis, hypoglycemia, hyperlactatemia, and hyperfibrinogenemia occur and the severity of these alterations determine the prognosis of the diarrheic calves [21,22].

In view of such consideration, the aim of this study was to evaluate the possible changes in lipid variables, alkaline phosphatase (ALP), and bilirubin values to estimate a possible liver failure in neonatal calves affected by diarrhea.

## Materials and Methods

### Ethical approval

This study was conducted after obtaining approval from the Kafkas University Animal Experiments Local Ethics Committee (KAU HADYEK-Submission 2016/77).

### Animals

In this study, 25 calves with diarrhea that were brought to Department of Internal Medicine, School of Veterinary Medicine, Kafkas University and 10 clinically healthy calves aged 1-30 days old were used. The etiologic diagnosis was not considered in diarrheic calves, and routine clinical examination was performed in all animals. Blood samples were collected into tubes with ethylenediaminetetraacetic acid and then centrifuged at 3000 rpm for 10 min. Plasma samples were then kept at −20°C until analysis.

### Biochemical analysis

Plasma levels of triglycerides, total cholesterol, HDL-C, ALP, direct and total bilirubin were determined colorimetrically (Epoch, BioTek, USA) using commercial kits (DDS-Turkey). VLDL-C and LDL-C levels were calculated according to the Friedewald formula [23] LDL-C = (Total cholesterol) − (HDL-C) − (Triglycerides/5). VLDL-C was calculated as [VLDL-C (mg/dL) = Triglyceride (mg/dL)/5] in case triglyceride (mg/dL) was <400 mg/dL.

### Statistical analysis

SPSS [24] for Windows 20.0 was used for the statistical analyses. The distribution of the data obtained from the groups was shown as normal distribution according to the Kolmogorov–Smirnov test. Therefore, Student’s t-test was used to compare the differences of the values obtained diarrheic calf and control group. The levels of significance accepted were p<0.05, p<0.01 and p<0.001, as stated below.

### Results

The plasma concentrations of ALP (p<0.05), total and direct bilirubin, triglyceride and VLDL-C (p<0.01) were significantly increased, whereas total cholesterol, HDL-C and LDL-C (p<0.01) values were significantly decreased in diarrheic calves compared to those of the control group (Table-1).

**Table-1:** Mean and the standard error of the mean (x¯±SE) values for ALP, direct and total bilirubin, triglycerides, total cholesterol, HDL-C, VLDL-C and LDL-C of neonatal calves with diarrhea.

Parameters	Control n=10	Infected n-25	p
ALP (U/L)	73.56±3.52	82.72±2.20	<0.05
Total bilirubin (mg/dL)	0.13±0.02	0.24±0.01	<0.01
Direct bilirubin (mg/dL)	0.041±0.003	0.119±0.010	<0.01
Triglyceride (mg/dL)	23.76±0.99	33.68±1.13	<0.01
Total cholesterol (mg/dL)	87.24±2.88	55.61±1.49	<0.01
HDL-C (mg/dL)	45.48±2.65	34.67±1.11	<0.01
VLDL-C (mg/dL)	4.75±0.20	6.74±0.23	<0.01
LDL-C (mg/dL)	37.01±3.97	14.20±1.42	<0.01

ALP=Alkaline phosphatase, HDL-C=High density lipoprotein-cholesterol, VLDL-C=Very low density lipoprotein-cholesterol, LDL-C=Low density lipoprotein-cholesterol, SE=Standard error

## Discussion

Newborn calves are susceptible to neonatal calf diarrhea especially during their first 28 days of life. Neonatal diarrhea, or calf scours, is a common disease in calves, and still a major cause of productivity and economic loss to cattle producers worldwide [11]. It causes by infectious and noninfectious factors [4]. The diarrheic calf becomes dehydrated and suffers from electrolyte loss and acidosis. In addition to its effects on gastrointestinal system, it also effects other systems or organ functions such as lungs, kidneys, and liver [25].

Plasma lipids and lipoproteins are synthesized by the liver and released into the bloodstream; determination of plasma lipid and lipoprotein concentrations has been reported to be helpful in eliciting hepatic dysfunction [26,27].

Lipids are molecules that are structurally and functionally involved in biological membranes and stored as triglycerides in the fatty tissue and liver. Although plasma triglyceride concentrations are reported to variable in acute cases [16,28]. However, hepatic triglyceride production is always elevated due to an increase in free fatty acids release from lipid deposits. Degradation of lipid deposits induced by cytokines such as tumor necrosis factor α (TNF-α) and interleukin 1 (IL-1) and increased *de novo* fatty acid synthesis in the liver effects plasma free fatty acids levels [29]. Plasma triglyceride levels are associated with enzymes that are effective in hepatic triglyceride production and with VLDL secretion. It is also depend on the effectiveness of mechanisms that remove VLDL triglycerides such as lipoprotein lipase mediated lipolysis and the uptake of residues by the tissues. Triglyceride metabolism has reported to be normal in many acute cases, and plasma triglyceride levels found to be normal or low [30]. This condition is usually observed after myocardial infarction or postoperatively. However, plasma triglyceride level is increased in sepsis because of suppressed plasma lipoprotein lipase activity. It has been reported that lipopolysaccharide (LPS) and endotoxins in the wall structure of Gram-negative bacteria such as *E. coli* and *Salmonella* spp. increase serum triglyceride levels due to activation of cytokines (TNF-α or IL-1β) [31-33]. In our study, triglyceride levels were also increased in calves with diarrhea and it was thought that the increase in triglyceride level might possibly result from cytokine activation reported in diarrheic calves [33]. Cholesterol levels are known to increase in nephrotic syndrome or protein-losing nephropathy, hypothyroidism, acute pancreatitis and cholestasis, while decreasing in protein-losing enteropathy, hypoadrenocorticism and in acute inflammatory responses, associated with the production of proinflamatoric cytokines such as TNF-α or IL-1β [12,16]. In our study, total cholesterol levels were also low in diarrheic calves; most probably occurs due to inflammatory reaction and/or protein-losing enteropathy.

The lipolysis of fat tissue along with increase in the concentration of *de novo* synthesized fatty acids during inflammation and infection increase triglyceride production in the liver and consequently increases VLDL secretion. This increase in VLDL concentration was reported to have a protective effect through binding and thus detoxification of LPS in the wall structure of Gram-negative bacteria [28]. Normally, LDL-C is a lipoprotein that transports phospholipids and fat-soluble vitamins from the liver to the tissues and is very important in the pathogenesis of cardiovascular diseases. HDL-C transports cholesterol from peripheral tissues to the liver and it has anti-inflammatory effect. It has been reported that the concentration of HDL-C decreases during inflammatory responses [16]. A significant increase in VLDL-C levels and a decrease in HDL-C and LDL-C levels were also determined in our study group compared to those of the control group. It is likely that the increase in VLDL-C levels may occurs due to an increase in triglyceride levels in liver [16]. Furthermore, decrease in HDL-C and LDL-C levels may occur due to decrease in the synthesis of cholesterol, involved in the structure of these lipoproteins, due to liver damage [34].

ALP activity is known to increases in cholestasis, bone degradation, altered hepatocellular circulation and during the stress. Total bilirubin increases in hemolysis, primary liver injury and bile duct inflammation, while direct bilirubin increases in hepatic diseases and bile duct inflammation [15,35]. In studies, increases in serum creatinine, glucose, total bilirubin, aspartate aminotransferase, alanine aminotransferase and gamma glutamyltransferase levels were reported in calves with diarrhea [22,36,37]. In this study, significant increases in ALP, total and direct bilirubin levels were determined in the study group compared to the control group, and this was thought to be liver and bile duct damage developed in calves with diarrhea.

## Conclusion

The results of this study have shown that significant increases in ALP, total bilirubin, triglyceride, and VLDL-C values were determined whereas, significant decreases in total cholesterol, HDL-C and LDL-C values were observed in diarrheic neonatal calves. It is possible to say that liver function deteriorates, and therefore the lipid profile is affected negatively in calves with diarrhea.

## Authors’ Contributions

This work was carried out in collaboration between all authors. KB, OM and HIG: Designed the experimental procedures. KB, OM and GG: Conducted the research work. OM, MO, SM: Helped in laboratory analysis. KB, OM, HAD: Prepared figures, tables, revised and submitted the manuscript. All authors read and approved the final manuscript.
